# Awake craniotomy for brain tumor resection: anesthetic management and clinical experience from a high complexity hospital in Latin America

**DOI:** 10.1186/s12871-025-03393-4

**Published:** 2025-10-22

**Authors:** David Esteban Eraso-Bolaños, Laura Libreros-Peña, Claudia Y. Komaromy-Obando, Isabel C. Soto-González

**Affiliations:** 1 Department of Surgical Clinics, Anesthesiology Residency Program, Pontificia Universidad Javeriana Cali, Cali, Colombia; 2 Departament of Anesthesiology, Centro Médico Imbanaco, Cali, Colombia

**Keywords:** Awake craniotomy, Brain tumor resection, Anesthetic management, Monitored anesthesia care, Surgical outcomes

## Abstract

**Background:**

Awake craniotomy is the gold standard for resecting brain tumors in eloquent areas. This technique requires tailored anesthetic management to ensure patient safety and cooperation. We aimed to describe clinical characteristics, anesthetic management, and intraoperative outcomes in patients undergoing awake craniotomy at a high-complexity hospital in southwestern Colombia.

**Methods:**

We conducted a retrospective case series. Sociodemographic, clinical, and intraoperative data were obtained from electronic medical records. Descriptive statistics were used to summarize the findings.

**Results:**

Fifteen patients underwent awake craniotomy with monitored anesthesia care using dexmedetomidine and remifentanil. All patients received a scalp block, non-opioid analgesics, and antiemetic prophylaxis. The most frequent adverse event was transient bradycardia without hemodynamic instability. No respiratory or neurological complications occurred. All procedures were successfully completed.

**Conclusions:**

Awake craniotomy under monitored anesthesia care with dexmedetomidine and remifentanil was feasible, safe, and well tolerated in this cohort. The absence of serious complications highlights the value of structured anesthetic protocols and trained multidisciplinary teams. This experience provides reference for the adoption of similar strategies in hospital settings across Latin America, where implementation of awake craniotomy remains limited.

## Introduction

Awake craniotomy is a neurosurgical technique that allows real time assessment of neurological function during tumor resection, particularly when lesions are located in eloquent cortical areas [1]. The main objective is to maximize the extent of resection while preserving functions such as speech, motor control, and sensory processing. Successful performance of this surgical intervention requires meticulous anesthetic planning and patient cooperation [1–3].

Anesthetic management plays a central role in ensuring patient comfort, key objectives include maintaining spontaneous ventilation, enabling adequate cooperation during cortical mapping, avoiding adverse events such as respiratory depression, nausea, vomiting, or seizures [1–3]. Monitored anesthesia care (MAC) combined with regional scalp block is commonly used; therefore, the selection of anesthetic agents with favorable profiles is essential [4]. Dexmedetomidine provides sedative, anxiolytic, and analgesic effects while preserving respiratory drive, making it. particularly suitable for awake craniotomy. When combined with remifentanil, it allows precise titration of sedation and analgesia through synergistic effects [5–7].

Brain tumors located near or within eloquent regions represent clinical challenge, as surgical resection must balance oncological control with functional preservation. Awake craniotomy has therefore become the gold standard, enabling accurate intraoperative cortical mapping and direct patient participation [4, 7].

Despite increasing global experience, there is limited research from low-middle-income countries (LMICS) [8–10]. The present study aims to describe our clinical experience with awake craniotomy following a multimodal strategy in a high-complexity Latin American Hospital, intending to contribute regional evidence and highlight the feasibility of implementing similar strategies in LMICs.

## Methods

### Design

We conducted a retrospective observational descriptive study of adult patients (> 18 years), diagnosed with supratentorial tumors who underwent awake craniotomy for tumor resection at Clinica Imbanaco in Cali, Colombia between January and December 2024.

The Institutional Review Board of Pontificia Universidad Javeriana Cali and Clinica Imbanaco respectively approved the protocol of this study (Act No. 11 of 2024 under the code CEI-975). Given the retrospective nature of this research, informed consent was not required as it was classified as risk-free according to national resolution (No. 008430 of 1993, article 11, numeral A) of the Ministry of Health and Social Protection of Colombia.

## Interventions

General interventions include preoperative evaluation and patient preparation, including neuropsychological assessment of cognitive components, intraoperative anesthetic management and postoperative care carried out as follows:

### Preoperative assessment

All patients underwent pre-anesthetic consultation including physical, and neuropsychological evaluations. The assessments focused on airway examination and neurocognitive functions (language, memory, attention, motor and executive domains). Patients were counseled regarding the procedure, particularly the phases during which they would remain awake.

#### Intraoperative management

 Standard monitoring included electrocardiography (ECG), pulse oximetry, capnography, and non-invasive or invasive arterial blood pressure measurement. Intraoperative neuromonitoring was performed using cortical mapping with somatosensory and motor evoked potentials. Real-time neurological assessments during surgery focused on language and motor tasks coordinated with neurosurgical team to detect signs of deterioration.

Oxygen supplementation was provided via nasal cannula with exhaled CO2 monitoring (2–3 L/minute, titrated to patient needs). All patients received bilateral scalp block with 2–3 mL of local anesthetic (levobupivacaine 0.35%), prophylaxis against postoperative nausea and vomiting included dexamethasone (8-16 mg) and ondansetron (8 mg), with haloperidol selectively. Multimodal analgesia included a single dose of 1 gr of paracetamol and NSAID, unless contraindicated.

Once full monitoring is established, dexmedetomidine infusion was initiated at rates not exceeding 0.7 mcg/kg/h. Simultaneously, remifentanil was administered via target controlled infusion (TCI), using Minto pharmacokinetic model, titrated to effect-site concentrations of 0.5–2.5 ng/ml. Infusions were adjusted throughout the surgery according to the patients level of consciousness to ensure comfort, cooperation, spontaneous ventilation, and adequate conditions during each surgical stage (incision, craniotomy, cortical mapping, resection, and closure).

Based on pharmacokinetic properties, dexmedetomidine was discontinued approximately 20 min before the end of surgery, and remifentanil 5–7 min before.

#### Postoperative care

 Following surgery, patients were transferred to Neurosurgical Intensive Care Unit (NICU) for continuous neurological monitoring and postoperative neuroimaging to detect potential complications. Subsequently the patient is usually derived to the hospitalization unit hospital discharge.

### Variables and data collection

All data were retrospectively obtained from institutional electronic medical records and recorded in Microsoft Excel^®^. We included sociodemographic, preoperative and intraoperative clinical information.

Intraoperative complications were defined according to institutional protocol. Cardiovascular complications included hypotension (≥ 20% decrease in systolic blood pressure compared with baseline), hypertension (systolic blood pressure > 140 mmHg), tachycardia (heart rate > 100 bpm), and bradycardia (heart rate < 60 bpm). Respiratory complications included desaturation (SpO₂ <92%) and apnea (temporary absence of spontaneous breathing). Neurological complications included seizures (loss of consciousness, repetitive or rhythmic movements of the head or extremities, and/or rigidity) and lack of cooperation during neuropsychological testing.

### Statistical analysis

Qualitative variables were summarized using absolute and relative frequencies. For numerical variables, measures of central tendency and dispersion were chosen according to the distribution of the data. Normally distributed variables (e.g., age) were reported as mean ± standard deviation (SD), whereas non-normally distributed variables (e.g., tumor size) were reported as median and interquartile range (25th–75th percentiles). Only descriptive statistics were applied given the small sample size. Analysis was performed using Stata 18, (Stata Corp 2023).

For the description of categorical variables, absolute and relative frequencies were used to represent the distribution of the categories in a clear and understandable manner. In the case of numerical variables, robust measures of central tendency and dispersion were used, specifically the median and the interquartile range (IQR), since these allow a better representation of the data when the assumption of normality is not met.

## Results

 We included fifteen patients during the study time. The mean age was 47.9 years. Most patients were male (60%) and of Latin-American mestizo ethnicity (86.67%). Regarding comorbidities, (66.7%) had a history of epilepsy as the sole diagnosis, while the remaining individuals presented epilepsy in association with other conditions (e.g., hypothyroidism, hypertension, HIV, latent tuberculosis). Additional reported comorbidities included fibromyalgia, ovarian cancer, Diabetes Mellitus, and glaucoma. Only one patient reported no comorbidities at all (Table 1).

**Table 1:** Demographic and clinical profile of patients

Variable		*N* = 15
Gender		
Male		9 (60%)
Female		6 (40%)
Age (years)		47.93 (12)
Ethnicity		
Afro-descendant		1 (6.7%)
White		1 (6.7%)
Latin-American Mestizo		13 (86.7%)
Comorbidities		
Epilepsy		10 (66.7%)
Hypertension		3 (20%)
Hypothyroidism		2 (13.3%)
Lidocaine allergy		1 (6.7%)
Ovarian cancer		1 (6.7%)
Diabetes		1 (6.7%)
Endometriosis		1 (6.7%)
Fibromyalgia		1 (6.7%)
Glaucoma		1 (6.7%)
Latent tuberculosis		1 (6.7%)
HIV		1 (6.7%)
No		1 (6.7%)
	*n* (%); Mean (SD)	

There was a varied distribution in the location of the brain tumors, with a predominance of the right frontal (33.3%) and left frontal (26.7%) regions. The median size of the tumors was 29 mm, with a range that varied between 18 and 68 mm. Regarding preoperative neurological status, most patients (86.7%) had an optimal level of consciousness (Glasgow 15), while the remaining 13.3% showed a slight decrease in the scale with a score of 14 (Table 2).

**Table 2:** Brain tumor distribution and preoperative neurological condition

Variable	*N* = 15
Tumor location	
Right frontal lobe	5 (33.3%)
Left frontal lobe	4 (26.7%)
Left parietal lobe	1 (6.7%)
Right temporal lobe	2 (13.3%)
Left temporal lobe	3 (20%)
Tumor size (mm)	29 (P25–P75)
Preoperative neurological condition	
Glasgow 14	2 (13.3%)
Glasgow 15	13 (86.7%)
*n* (%); Median (P25-P75)	

All patients underwent awake craniotomy under monitored anesthesia care. Scalp block was performed in all cases. The anesthetic regimen in all patients included continuous infusions of dexmedetomidine and remifentanil with variable target doses. Dexmedetomidine was administered at an infusion range between 0.1 and 0.5 mcg/kg/h combined with remifentanil at site effect concentrations between 0.5 and 2.0 ng/ml using Minto pharmacokinetic model for Target-controlled infusion (TCI), with two patients requiring higher remifentanil targets up to 2.5 ng/ml.

Regarding analgesic management, all patients received at least one non-opioid analgesic: 40% received a combination of paracetamol and ketorolac, 26.67% received paracetamol and parecoxib, and 20% received paracetamol alone.

All patients received antiemetic prophylaxis with dexamethasone and ondansetron. In selected patients, haloperidol was included as part of the regimen for the prevention of postoperative nausea and vomiting. Additional medications were administered according to the anticipated intraoperative and postoperative needs. levetiracetam and lacosamide were used in most cases as seizures prophylaxis according to previously taken antiepileptic drugs (AEDs). Tranexamic acid was administered in several cases as patient blood management (PBM) strategy to minimize intraoperative and postoperative bleeding and reduce the need for transfusions. These medications were combined in various protocols tailored to individual patient characteristics and surgical considerations (Table 3).

**Table 3:** Intraoperative clinical characteristics

Variable	*N* = 15
Scalp block	
Yes	15 (100%)
Anesthetic technique used	
Monitored Anesthesia Care (MAC)	15 (100%)
Anesthetic agents administered	
Dexmedetomidine	15 (100%)
Remifentanil	15 (100%)
Dexmedetomidine (mcg/kg/h)	
0.1–0.3	1.0 (6.7%)
0.1–0.5	9 (60%)
0.1–0.7	1 (6.7%)
0.2–0.5	1 (6.7%)
0.3–0.5	3 (20%)
Remifentanil (ng/ml)	
0.5-2	1 (6.7%)
0.6-2	1 (6.7%)
1–2	8 (53.3%)
1-2.2	1 (6.7%)
1	3 (20%)
2-2.5	1 (6.7%)
Analgesic agents administered	
Ketorolac	6 (40%)
No	2 (13.3%)
Paracetamol	13 (86.7%)
Parecoxib	4 (26.7%)
Other pharmacological agents used	
Tranexamic acid	14 (93.3%)
Dexamethasone	15 (100%)
Haloperidol	2 (13.3%)
Lacosamide	4 (26.7%)
Levetiracetam	10 (66.7%)
Ondansetron	15 (100%)
Surgery duration (minutes)	247.27 (74.10)
Anesthesia duration (minutes)	341.13 (78.93)
*n* (%); Mean (SD)	

Intraoperative complications were infrequent in this cohort. Transient bradycardia occurred in 8 of the 15 patients. The minimum recorded heart rates ranged from 42 to 57 beats per minute without hemodynamic impact and need of vasopressor support. No respiratory or neurological complications were reported intraoperatively, and no failures of the awake craniotomy protocol were recorded. All procedures were completed successfully as planned (Table 4).

**Table 4:** Intraoperative complications

Variable	*N* = 15
Intraoperative cardiovascular complications	
No	6 (40%)
Yes	9 (60%)
Intraoperative bradycardia	
Minimum HR 42 bpm	1 (6.7%)
Minimum HR 47 bpm	1 (6.7%)
Minimum HR 50 bpm	1 (6.7%)
Minimum HR 53 bpm	1 (6.7%)
Minimum HR 55 bpm	2 (13.3%)
Minimum HR 56 bpm	1 (6.7%)
Minimum HR 57 bpm	1 (6.7%)
Intraoperative respiratory complications	
No	15 (100%)
Intraoperative neurological complications	
No	15 (100%)
Failed awake craniotomy	
No	15 (100%)
*n* (%)	

## Discussion

### Principal findings

In our experience, all procedures were successfully completed under regional anesthesia (scalp block) and monitored anesthesia care with a combination of dexmedetomidine infusion and remifentanil delivered using a TCI system, without conversion to general anesthesia. Bradycardia was the most frequent cardiovascular event, likely related to the synergistic effect of the agents used. These episodes were transient and did not result in hemodynamic instability and required neither vasopressor support nor modifications to the anesthetic strategy. No respiratory or neurological complications were observed. This emphasizes the importance of careful titration, continuous monitoring, and trained teams to maintain patient safety throughout the different stages of awake craniotomy.

### Research in context

Our results are consistent with previous reports supporting the use of dexmedetomidine as part of anesthetic strategies for awake craniotomy. Goettel et al. conducted a randomized controlled trial comparing dexmedetomidine with propofol–remifentanil for monitored anesthesia care in patients undergoing supratentorial tumor resection. No differences were found regarding sedation effectiveness or mapping quality, although dexmedetomidine was associated with fewer respiratory adverse effects and lower heart rate, without differences in the need for [7].

In addition, the use of dexmedetomidine in neurosurgical patients might have neuroprotective advantages with minimum impact in neuronal function, hemodynamic stability, and respiratory drive, allowing for stable intraoperative conditions ensuring patient cooperation and enhancing safety [5, 7].

Anesthetic management in awake craniotomy does not have a standardized protocol, techniques to provide adequate analgesia and sedation levels can vary greatly among centers. The choice of sedative agents is based on several factors such as safety, availability and predictability of their effects on consciousness and intraoperative cortical mapping electrocorticography. Jose et al. described the use of Propofol, Remifentanil and Dexmedetomidine alone or in combination due to their pharmacokinetic profiles allowing rapid recovery and smooth emergence [8].

Accordingly, to the sedative agents used in our study, it has been reported that Remifentanil for awake craniotomy is commonly used at 0.05 to 0.1 mcg/kg/min or between 2 and 3 ng/ml via TCI and Dexmedetomidine initially administered with an initial bolus of 1 mcg/kg for 10 min followed by an infusion from 0.2 to 0.7 mcg/kg/h [9]. Furthermore, Izzi et al. in 2024 also described the safety of using the combination of dexmedetomidine infusion at low dosages 0.7 mcg/kg/h and Remifentanil via TCI to the site effect with the Minto model between 0.5 and 1 ng/ml for conscious sedation in awake craniotomy [6].

Thus, the optimal combination of anesthetic agents used in AC continues to be a subject of debate. Aghajanian et al. conducted a systematic review highlighting the great variability in anesthetic protocols used in MAC for AC. Future research is required to draw definitive conclusions [10].

Maintaining airway patency and avoiding respiratory depression and hypercapnia are essential in this type of neurosurgical intervention. McAuliffe et al. conducted a retrospective study including 55 patients who received scalp block and sedation with Dexmedetomidine infusion. This approach offered comfort and some degree of anxiolysis to patients undergoing awake craniotomy, allowing successful intraoperative neurological monitoring. No adverse events were reported related to critical airway manipulation or the need for general anesthesia [11]. The aforementioned is consistent with our results, where there was no need to intervene with the airway, and each procedure was performed successfully.

Even though none of the patients included in our cohort presented intraoperative seizures, in the context of awake craniotomy, this neurological complication remains a significant concern due to cortical stimulation and tumor manipulation. The use of AEDs (e.g. Levetiracetam) for seizure control and prophylaxis has demonstrated efficacy and better tolerability according to previous studies, increasingly favored over phenytoin due to its low incidence of side effects, and lack of hepatic enzyme induction and minimal pharmacologic interactions being ideal in patients with a high burden of comorbidities or polypharmacy [12].

Although the implementation of awake craniotomy for removing cerebral tumors in Latin American and LMICs remains full of challenges, our study, carried out in a high complexity hospital in Colombia, allows us to compare its favorable results with other institutions from LMICs. Albuquerque et al. conducted a prospective observational study including 51 patients from 18 to 70 years in Brazil, emphasizing the importance of a dedicated multidisciplinary team planning and performing these procedures on a regular basis to obtain good functional outcomes [13].

Although most available reports on awake craniotomy come from high-income countries, evidence from Latin America and other LMICs is steadily increasing.In Brazil, a prospective observational study emphasized the role of a dedicated multidisciplinary team and regular performance of these procedures to ensure good outcomes [13, 14]. Our results add to this regional experience and show that awake craniotomy can also be safely performed in Colombia. Beyond Latin America, additional reports from India, Egypt, and Pakistan further confirm the feasibility and safety of this approach in different LMIC contexts, underscoring the global relevance of adapting anesthetic protocols to resource-limited settings [15].

### Limitations and strengths

The main limitation of our study is the retrospective nature of the study and the small sample size, and single center setting, which limit generalizability. Besides, our study was conducted at Clinica Imbanaco, which is one of the few high complexity institutions in the country that performs awake craniotomy and provides health care to a significant area of the southern territory of Colombia, a region primarily inhabited mainly by indigenous, afro descendant and mestizo populations. The low number of cases reflects the limited frequency with which this procedure is performed, even in high complexity centers.

By sharing our experience, our findings provide valuable evidence on performing awake craniotomy in LMICs. Moreover, this experience may serve as reference for other institutions in Latin America aiming to adopt similar protocols highlighting the absence of serious intraoperative complications.

### Future implications

For anesthesiologists and neurosurgical teams, this experience highlights the critical role of perioperative planning tailored to patient needs, with a focus on the safety and efficacy of regional anesthesia techniques and monitored anesthesia care. Future research should include prospective, multicenter studies with larger and more diverse populations to strengthen the evidence base. It will also be relevant to explore correlations between intraoperative spectrographic data and clinical variables during surgery that may allow assessment of long term functional, cognitive and quality of life outcomes, ideally carried out in comparable hospital settings.

## Conclusion

The present experience highlights the importance of trained teams, well-defined protocols and anesthetic strategies adapted to the local context. Awake craniotomy was successfully performed in all patients using the combination of dexmedetomidine and remifentanil, without conversion to general anesthesia, cardiovascular interventions or respiratory or neurological complications. These findings may contribute to building regional references and support the safe adoption of awake craniotomy strategies in similar clinical settings.

## Data Availability

The datasets analyzed during the current study and that support the findings of this study are available from Clínica Imbanaco, but restrictions apply to the availability of these data, due to internal privacy policies. Data are however available from David Eraso Bolaños upon reasonable requests and with permission of Clínica Imbanaco (davidee93 @javerianacali.edu.co).

## Abbreviations

MAC: Monitored Anesthesia Care
LMIC: Low- and Middle-Income Country
TCI: Target-Controlled Infusion
ECG: Electrocardiogram
AED: Antiepileptic Drug
PBM: Patient Blood Management
NICU: Neurosurgical Intensive Care Unit
HR: Heart Rate
SpO₂: Peripheral capillary oxygen saturation

## References

[1] Kulikov A, Lubnin A. Anesthesia for awake craniotomy. Vol. 31, current opinion in anaesthesiology. Lippincott Williams and Wilkins; 2018. pp. 506–10. 10.1097/ACO.0000000000000625 29994938

[2] Akavipat P, Sookplung P, Lekprasert V, Kasemsiri C, Lerdsirisophon S. Dexmedetomidine for awake craniotomy: systematic review and meta-analysis. J Clin Neurosci. Churchill Livingstone; 2024. Volume 127. 10.1016/j.jocn.2024.110765. 10.1016/j.jocn.2024.110765 39079421

[3] Izzi A, Mincolelli G, D’Onofrio G, Marchello V, Manuali A, Icolaro N et al. Awake craniotomy in conscious sedation: the role of A2 agonists. Brain Sci. 2024;14(2). 10.3390/brainsci14020147 PMC10886693 38391722

[4] Zhang K, Gelb AW. Awake craniotomy: Indications, benefits, and techniques. Colombian Journal of Anesthesiology. Volume 46. Lippincott Williams and Wilkins; 2018. pp. 46–51.

[5] Lin N, Vutskits L, Bebawy JF, Gelb AW. Perspectives on Dexmedetomidine use for neurosurgical patients. Journal of Neurosurgical Anesthesiology. Volume 31. Lippincott Williams and Wilkins; 2019. pp. 366–77. 10.1097/ANA.0000000000000554 30363004

[6] Goettel N, Bharadwaj S, Venkatraghavan L, Mehta J, Bernstein M, Manninen PH. Dexmedetomidine vs propofol-remifentanil conscious sedation for awake craniotomy: A prospective randomized controlled trial. Br J Anaesth. 2016;116(6):811–21. 27099154 10.1093/bja/aew024

[7] Hussein K, Moore M. Anaesthesia Management for Awake Craniotomy [Internet]. 2022. Available from: https://resources.wfsahq.org/anaesthesia-tutorial-of-the-week/

[8] Jose GRB, Legaspi GD, Ibale MGD, Duñgo ABC. Awake craniotomy: nuts and bolts. Int Anesthesiol Clin. 2023;61(3):8–12. 37243429 10.1097/AIA.0000000000000408

[9] Ozlu O, Sanalbas S, Yazicioglu D, Utebey G, Baran I. Sedation and regional anesthesia for deep brain stimulation in parkinson’s disease. J Anesthesiology. 2014;2014:1–6.

[10] Aghajanian S, Naeimi A, Mohammadifard F, Mohammadi I, Rajai Firouzabadi S, Baradaran Bagheri A, et al. Efficacy and safety of anesthetic agents in awake craniotomy using monitored anesthesia care protocol: a systematic review and meta-analysis. Neurosurg Rev. 2025;48(1):57. 39815114 10.1007/s10143-025-03176-y

[11] McAuliffe N, Nicholson S, Rigamonti A, Hare GMT, Cusimano M, Garavaglia M, et al. Awake craniotomy using Dexmedetomidine and scalp blocks: a retrospective cohort study. Can J Anesth. 2018;65(10):1129–37. 29978278 10.1007/s12630-018-1178-z

[12] Iuchi T, Kuwabara K, Matsumoto M, Kawasaki K, Hasegawa Y, Sakaida T. Levetiracetam versus phenytoin for seizure prophylaxis during and early after craniotomy for brain tumours: A phase II prospective, randomised study. J Neurol Neurosurg Psychiatry. 2015;86(10):1158–62. 25511789 10.1136/jnnp-2014-308584

[13] Albuquerque LAF, Macêdo Filho LJM, Borges FS, Pessoa FC, Diógenes GS, Rocha CJV, et al. Awake craniotomy for diffuse low grade gliomas in a resource limited setting: lessons learned with a consecutive series of 51 surgeries. World Neurosurg. 2023;177:e563–79. 37385443 10.1016/j.wneu.2023.06.096

[14] Figueredo LF, Shelton WJ, Tagle-Vega U, Sanchez E, de Macedo Filho L, Salazar AF, et al. The state of Art of awake craniotomy in Latin American countries: a scoping review. Journal of Neuro-Oncology. Volume 164. Springer; 2023. pp. 287–98. 10.1007/s11060-023-04433-0 37698707

[15] Bharadwaj HR, Awuah WA, Adebusoye FT, Tan JK, Ali SH, Pacheco-Barrios N et al. Awake craniotomies in South america: Advancements, challenges, and future prospects. J Cent Nerv Syst Dis. 2024;16. 10.1177/11795735241238681 PMC10938621 38487717

